# Comparative analysis of defensin expression in *Hyalomma asiaticum* ticks exposed to *Staphylococcus aureus* and *Escherichia coli*

**DOI:** 10.3389/fvets.2026.1902523

**Published:** 2026-08-24

**Authors:** Zhen Ma, Depeng Yang, Tingxiang Luo, Sen Yang, Wenxiu Hu, Qiya Wu, Zhengxiang Hu, Yongchang Li, Yang Zhang, Wei Zhang, Bayinchahan Gailike, Qingyong Guo, Xiaoyun Mi, Ercha Hu, Caishan Li

**Affiliations:** 1 College of Veterinary Medicine, Xinjiang Agricultural University, Urumqi, Xinjiang Uygur Autonomous Region, China; 2 College of Grassland Science, Xinjiang Agricultural University, Urumqi, Xinjiang, Uygur Autonomous Region, China; 3 Xinjiang Key Laboratory of New Drug Research and Development for Herbivorous Animals, Xinjiang, Uygur Autonomous Region, China; 4 Xinjiang Regional Key Laboratory of Clinical Veterinary Medicine Research, Xinjiang, Uygur Autonomous Region, China; 5 Urumqi Field Scientific Observation and Research Station for Animal Diseases, Ministry of Agriculture and Rural Affairs, Xinjiang, Uygur Autonomous Region, China; 6 Veterinary Medicine Postdoctoral Research Station of Xinjiang Agricultural University, Urumqi, Xinjiang, Uygur Autonomous Region, China; 7 Xinjiang Key Laboratory of Animal Disease Research, Urumqi, Xinjiang, Uygur Autonomous Region, China

**Keywords:** bacterial challenge, comparative transcriptomics, defensin, *Hyalomma asiaticum*, innate immunity

## Abstract

**Background:**

*Hyalomma asiaticum*, which acts as a vector of zoonotic pathogens, relies on its innate immune system for defense.

**Methods:**

Unfed adult female *H. asiaticum* ticks were injected with *Escherichia coli* (Gram-negative) or *Staphylococcus aureus* (Gram-positive), with PBS-injected ticks as controls. After 24 h, RNA sequencing (RNA-Seq) was performed to compare transcriptomic profiles. Differential expression analysis, KEGG pathway enrichment, and phylogenetic characterization of defensin genes were conducted. RT-qPCR was used to validate selected defensin expression levels.

**Results:**

Transcriptome assembly yielded 195,132 unigenes. Principal component analysis (PCA) clearly separated the PBS control, *E. coli*-challenged (EC), and *S. aureus*-challenged (SA) groups. A total of 4,294 and 4,582 differentially expressed genes (DEGs) were identified in the EC and SA groups, respectively, compared to the PBS control. KEGG pathway enrichment analysis revealed that the MAPK signaling pathway and the Ras signaling pathway were commonly activated in both infection groups. In contrast, the lysosome pathway was significantly enriched among down-regulated DEGs in both EC and SA groups. Fifteen putative defensin genes were identified from the transcriptome, featuring conserved RVRR, CXXXC and CXC motifs. Several of these defensin isoforms were significantly up-regulated following bacterial challenge, indicating their involvement in the antibacterial response.

**Conclusion:**

This comparative transcriptomic study provides a comprehensive characterization of the defensin repertoire and immune signaling responses of *H. asiaticum* to Gram-negative and Gram-positive bacterial challenges. The shared activation of the MAPK pathway, together with significantly enrichment of Ras signaling pathways, reveals both common and distinct antibacterial defense strategies. The strong up-regulation of defensins and the down-regulation of lysosome highlight key transcriptional events. These findings offer a valuable resource for understanding tick innate immunity and may inform future interventions for tick-borne disease control.

## 1 Introduction

Ticks are obligate ectoparasitic arthropods that rely exclusively on vertebrate blood for survival (1, 2). Ticks are second only to mosquitoes as vectors of pathogens in humans or livestock, and can transmit multiple pathogens comprising bacteria, fungi, viruses, protozoa, and nematodes. Diseases transmitted by ticks, such as Lyme disease, tick-borne encephalitis, human granulocytic anaplasmosis, and various rickettsioses, present a growing threat to public health and causing significant economic setbacks for livestock production worldwide (3–5).

Ticks generate corresponding immune responses under the stress of various pathogenic microorganisms (6, 7). Previous studies revealed that, like other invertebrates, ticks defend themselves with a primitive innate immune system consisting of humoral and cellular responses (8). The cell-mediated action is represented by phagocytosis, encapsulation and nodulation; while the humoral defense depends on a variety of humoral factors such as pattern recognition receptors, complement-related molecules, lectins, and antimicrobial peptides (AMPs) (9–11). Among tick antimicrobial substances, certain defensins exhibit potent bactericidal effects. Typically, ticks’ defensins contain six cysteine residues and are commonly expressed in the midgut following blood-feeding or pathogen invasion (12, 13). Tick defensins have a broad spectrum of antimicrobial profiles, which are primarily directed against Gram-positive bacteria, and few isoforms are also effective against Gram-negative bacteria (14–16).

Many stimulants related to microbial invasion, such as injury, infection, and blood-feeding, induce the secretion of these protective substances (17). Previous research indicated that following the injection of bacteria into tick species, defensins are expressed in an array of tick tissues, including the midgut, hemolymph, salivary glands, ovaries, and fat body (18, 19). The distribution pattern of defensins exhibits either tissue-specificity or a ubiquitous presence (20).

In this research, the objective was to induce an immune response in *Hyalomma asiaticum* by exposing it to both *Staphylococcus aureus* and *Escherichia coli* stress. Through this approach, the gene-level changes during the tick’s immune reaction were monitored. The primary goal was to identify and structurally elucidate the genes encoding antimicrobial effector molecules of *H. asiaticum* that are specifically up-regulated in response to particular pathogen invasions. To achieve this, a data-mining experiment was employed. By analyzing the gene expression data obtained from the bacterial-stressed ticks, candidate molecules that could be further investigated were identified. These findings are expected to potentially offer some insights on how ticks defend themselves against bacterial threats.

## 2 Materials and methods

### 2.1 Experimental samples, bacteria, and ethical statements

The *H. asiaticum* ticks used in the experiment were artificially reared and preserved at the College of Veterinary Medicine, Xinjiang Agricultural University. To maintain the optimal experimental environment, the ticks were kept in the laboratory following rearing and storage conditions in an ambient air temperature of 24 °C ± 1 °C, a relative humidity of 85% ± 5%, and a 14 h light and 10 h darkness photoperiod. The *S. aureus* (ATCC6538) and *E. coli* (CMCC44102) strains used in this study were commercially obtained (HaiBo Biotechnology, China). All animal procedures were performed in line with the guidelines in Document 2023025, approved by the Animal Ethics Committee of Xinjiang Agricultural University.

### 2.2 Microbial challenge experiments

**Figure 1:**
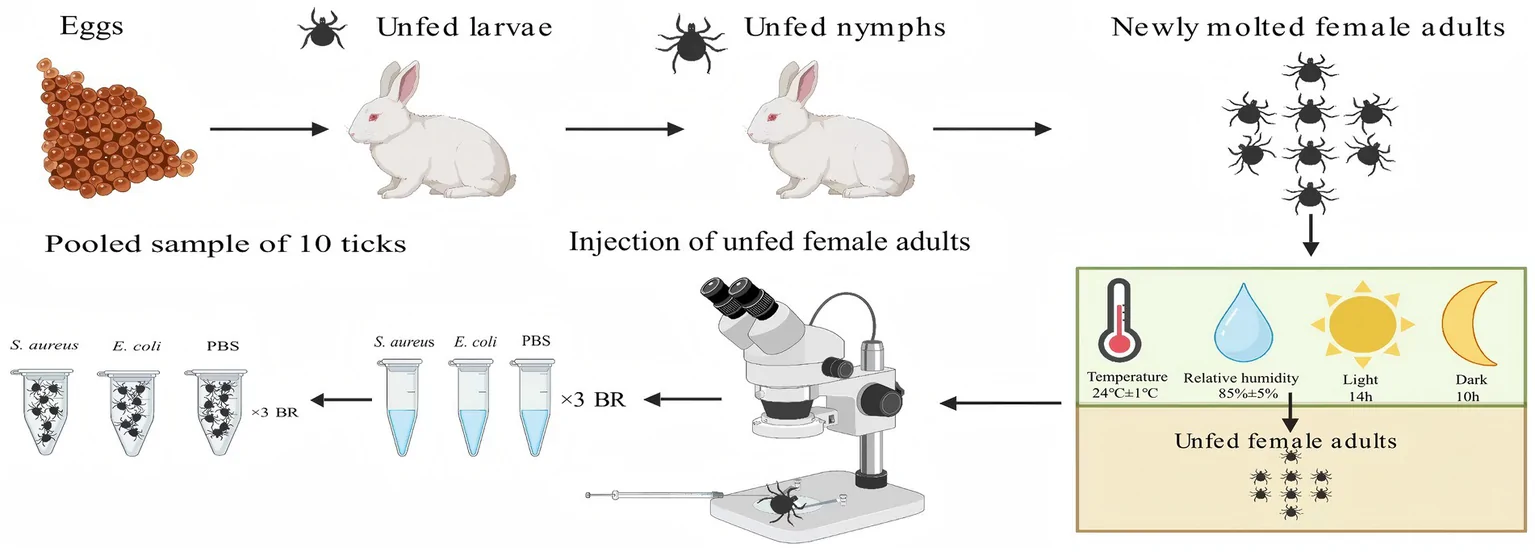
Schematic of tick experimental workflow. Unfed female *H. asiaticum* adults from a synchronized colony (reared at 24 °C, 85% humidity, 14 h:10 h light:dark) were injected with *S. aureus*, *E. coli* or PBS. At 24 h post-injection, ten ticks per group were pooled as one biological replicate (three replicates per condition) and snap-frozen for RNA-seq.

Unfed female ticks of similar body weight, from the same artificially reared batch at 2 weeks post-molting, were selected as the target samples for bacterial stress experiments. To minimize the impact of environmental microorganisms on the tick samples, their body surfaces were cleaned thoroughly. Briefly, each tick was immersed in sterilized PBS and subjected to shaking for 15 s, followed by transfer to a 75% ethanol solution for an additional 15 s with agitation. Subsequently, the ticks were rinsed three times with ddH_2_O with agitation applied during each rinse. Finally, the cleaned ticks were placed on sterilized absorbent paper for surface drying. *E. coli* and *S. aureus* were cultured to logarithmic phase in LB broth at 37 °C with shaking (200 rpm). Cells were harvested by centrifugation (4,000 rpm, 10 min, 4 °C), washed three times with sterile PBS, and resuspended in PBS. The viable CFU was determined by serial dilution and plating on LB agar. To determine the optimal injection dose and time point, pre-experiments with 10^5^–10^8^ CFU/500 nL per tick were performed; tick vitality was monitored at 24 h post-injection. Based on these pre-experimental results and previous transcriptomic studies, the combination of 10^7^ CFU/500 nL and the 24 h time point was selected, as it induced a clear immune response while maintaining >95% tick survival and high RNA integrity (19). Briefly, unfed female ticks were injected using a precision microsyringe (Hamilton Bonaduz, Switzerland) with a 10^7^ CFU/mL dilution of *S. aureus* or *E. coli* on the right side, between the fourth coxal segments, at a volume of 500 nL per tick. Immediately after completing the injection procedure, the injection site was sealed with a minimal amount of sterile Vaseline to prevent hemolymph leakage. After 24 h post-injection, ten individuals were randomly selected from each group to be pooled. The PBS-injected group was designated as the negative control to establish baseline gene expression profiles for comparative analysis. Each treatment group, including the negative control group, comprised 10 unfed female adult ticks. Three biological replicates were included for each group. Ticks were immediately transferred to freezing tubes and immersed in liquid nitrogen for snap-freezing to preserve RNA integrity prior to RNA-Seq (Figure 1).

### 2.3 RNA-Seq, RNA extraction, library preparation, and sequencing

Total RNA was extracted from tick samples using TRIzol Reagent (Invitrogen, California, USA) according to the manufacturer’s instructions. Following RNA extraction, DNA digestion was performed using DNase I (Thermo Fisher Scientific, Waltham, MA, USA) to eliminate any residual genomic DNA. The quality of the extracted RNAs was assessed by measuring the A260/A280 ratio with a Nanodrop™ OneC spectrophotometer (Thermo Fisher Scientific, Waltham, MA, USA). Additionally, RNA integrity was verified using either a Qsep 100 (BIOptic, New Taipei City, Taiwan, China) or an Agilent 2,100 (Agilent Technologies, Santa Clara, CA, USA) instrument, ensuring the high-quality and integrity of the RNA samples for downstream applications.

For the preparation of stranded RNA sequencing libraries, 1 μg of total RNAs was utilized following the manufacturer’s instructions for the KC-DigitalTM Stranded mRNA Library Prep Kit for Illumina® (Wuhan Seqhealth, China). This kit incorporates a unique molecular identifier (UMI) consisting of 8 random bases to label pre-amplified cDNA molecules, effectively eliminating duplication bias during the PCR and sequencing steps. Library products within the size range of 200–500 bps were enriched, accurately quantified, and subsequently sequenced on a NovaSeq 6,000 (Illumina, San Diego, CA, USA) in PE150 mode, ensuring high-throughput and accurate sequencing results.

### 2.4 *De novo* RNA-Seq data analysis

Raw sequencing data was first filtered by fastp (version 0.23.4), low-quality reads were discarded and the reads contaminated with adaptor sequences were trimmed. Clean reads were further treated with in-house scripts to eliminate duplication bias introduced in library preparation and sequencing. In brief, clean reads were first clustered according to the UMI sequences, in which reads with the same UMI sequence were grouped into the same cluster. Reads in the same cluster were compared to each other by pairwise alignment, and then reads with sequence identity over 95% were extracted to a new sub-cluster. After all sub-clusters were generated, multiple sequence alignment was performed to get one consensus sequence for each sub-clusters. After these steps, any errors and biases introduced by PCR amplification or sequencing were eliminated.

Then clean and deduplicated data were *de novo* assembled by Trinity (version 2.15.1) with default parameters (21). Sequencing reads were aligned back to the assembled transcripts for assessing the quality of the transcriptome assembly by using Bowtie2 (22). The longest transcripts of the same genes were screened as unigenes. For functional annotations of the unigenes, protein databases Nr, Uniprot, Pfam, eggNog, GO and KEGG were used. Unigenes differentially expressed between groups (*S. aureus, E. coli* and PBS) were identified using the edgeR package (version 3.12.1). A *p*-value cutoff of 0.05 and Fold-change cutoff of 2 were used to judge the statistical significance of gene expression differences. GO analysis and KEGG enrichment analysis for differentially expressed genes were both implemented by KOBAS software (version 2.1.1) with a *p*-value cutoff of 0.05 to judge statistically significant enrichment.

### 2.5 Defensin alignment and phylogenetic analysis

Putative defensin sequences from *H. asiaticum* were identified through a local BLAST alignment of the longest transcripts derived from RNA-Seq data against a well-curated database containing known tick defensins (Supplementary Table S1). To construct a comprehensive evolutionary framework, reference sequences from 19 tick species, such as *Ixodes scapularis*, *Dermacentor marginatus*, and *Ornithodoros turicata*, were sourced (23). Sequences that demonstrated significant homology were retained for further analysis.

All candidate defensin sequences were aligned using two distinct and complementary methods. First, ClustalW^1^ was employed with its default parameters, which included a gap opening penalty of 10 and a gap extension penalty of 0.1. Second, MAFFT v7.487 was utilized with the L-INS-i algorithm (24). This algorithm was chosen to optimize the alignment of structurally conserved regions within the sequences. The resulting alignments were then trimmed using trimAl v1.2 (25).

For the reconstruction of maximum-likelihood phylogenetic trees, IQ-TREE2 v2.2.2.6 was used (26). The best-fit substitution model, determined to be JTT + G4 through ModelFinder, was applied. Branch support was assessed using 1,000 ultrafast bootstrap replicates. The final tree topology was annotated and visualized in iTOL^2^ (27).

Multiple sequence alignments were manually examined and visualized using Jalview v2.11.5.1 (28). During this process, special attention was paid to conserved motifs, including the RVRR propeptide cleavage site and disulfide-bonded CXXXC/CXC motifs, as well as structural domains within the sequences.

### 2.6 Quantitative real-time PCR validation

To validate the RNA-seq-derived expression profiles, RT-qPCR analysis was conducted on defensin genes in tick samples. In brief, cDNA of each sample was synthesized using the Reverse Transcription System (Promega, Madison, USA). The cDNA was then used as template for RT-qPCR analysis, which was performed using the PrimeScript RT Reagent Kit (Takara Biotechnology, China) with gene-specific primers (Supplementary Table S2). Translation elongation factor EF-1 alpha (ELF1A) was used as an internal control. The relative expression levels of the immunity-related genes were calculated using the 2^^-△△^CT method and normalized to ELF1A.

## 3 Results

### 3.1 Transcriptome sequencing, assembly, functional annotation and global responses to bacterial challenge

Illumina sequencing of cDNA libraries from *H. asiaticum* females generated approximately 386 million high-quality clean reads across all samples (including PBS control, *E. coli* and *S. aureus* groups), with Q30 scores exceeding 97% and consistent GC content. *De novo* assembly using Trinity yielded 253,218 transcripts. To remove redundancy, these transcripts were further clustered into 195,132 non-redundant unigenes. The assembled transcripts had an average length of 788.09 bp, an N50 length of 1,306 bp, and a GC content of 47.13%. Correspondingly, the unigenes showed an average length of 641.88 bp, with an N50 length of 843 bp and a GC content of 46.76%. The assembly exhibited high integrity.

To comprehensively annotate gene functions, we aligned 195,132 Unigene sequences against six databases for functional annotation. Among these databases, Pfam provided the highest annotation rate, with 130,400 sequences annotated, while KEGG showed a relatively lower annotation rate, with only 20,378 sequences annotated. In total, 151,859 Unigene sequences were annotated across all databases (Supplementary Table S3). Gene Ontology (GO) functional annotation revealed that the majority of the annotated unigenes were distributed among three major categories: cellular component (integral component of membrane, nucleus and cytoplasm), biological process (dominated by DNA integration), and molecular function (nucleic acid binding) (Supplementary Figure S1). To further characterize the biological pathways associated with these sequences, the 20,378 unigenes that received KEGG annotations were subjected to pathway mapping. The most abundant sub-category was Metabolic pathways within the Metabolism branch, which accounted for 5,383 unigenes (26.42%). This was followed by Ribosome and Spliceosome in the Genetic Information Processing branch, with 1,635 unigenes (8.02%), and Thermogenesis in the Organismal Systems branch, comprising 789 unigenes (3.87%). These three pathways could potentially have a close association with the tick’s immune defense and digestion of blood meals, its maintenance of genetic information and protein synthesis, as well as its metabolic adaptation to nutrient utilization and prolonged starvation (Supplementary Figure S2). Notably, multiple signaling pathways closely associated with immune responses were identified in the KEGG annotation, including the Toll and Imd signaling pathway, Toll-like receptor signaling pathway, MAPK signaling pathway, JAK–STAT signaling pathway, and cytokine-cytokine receptor interaction. This may suggest the immune defense response mounted by ticks following bacterial infection, and the identification of these immune-related pathways also provides important clues for subsequent research on the innate immune defense mechanisms of ticks (Supplementary Table S4).

**Figure 2:**
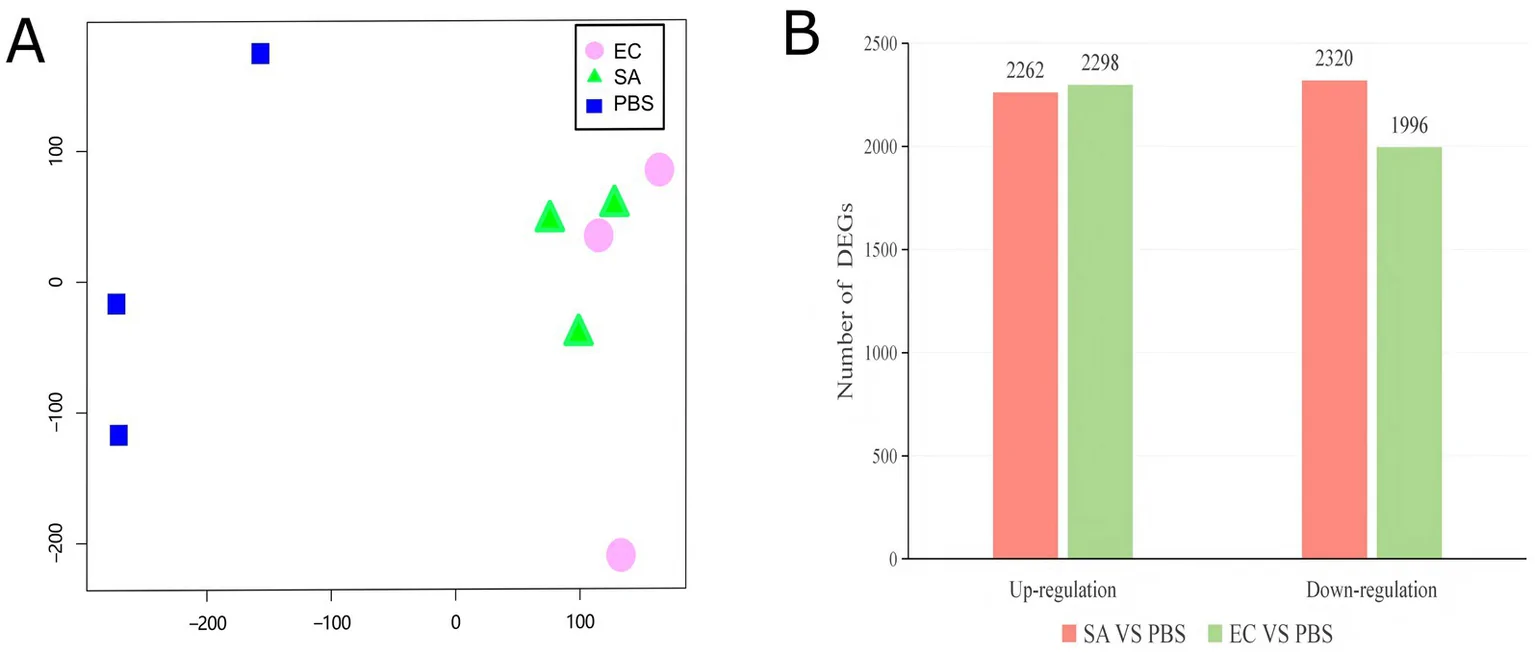
A principal component analysis (PCA) and DEGs identification. **(A)** PCA of transcriptome profiles from PBS-injected (PBS, blue squares), *E. coli*-challenged (EC, purple circles) and *S. aureus*-challenged (SA, green triangles) groups. Each dot represents a biological replicate (*n* = 3 per group). **(B)** Numbers of upregulated and downregulated DEGs in EC vs. PBS and SA vs. PBS comparisons (|log_2_ *FC*| > 1, *p* < 0.05). Total DEGs are indicated above each bar.

Principal component analysis (PCA) of gene expression profiles revealed clear separation among the PBS control, *E. coli*-challenged (EC), and *S. aureus*-challenged (SA) groups, indicating distinct transcriptomic reprogramming in response to different bacterial stimuli (Figure 2A). Comparative analysis identified a total of 4,294 differentially expressed genes (DEGs) in the EC group and 4,582 DEGs in the SA group compared to the PBS control (|log2 FoldChange| > 1; *p* < 0.05). Among these, compared to the PBS group, the EC group exhibited 2,298 up-regulated and 1,996 down-regulated DEGs, whereas the SA group showed 2,262 up-regulated and 2,320 down-regulated DEGs (Figure 2B). The transcriptome data, Trinity assembly sequences, and annotation table have been deposited in the NCBI Gene Expression Omnibus (GEO) database under the accession number GSE319477.^3^

### 3.2 Analysis and functional enrichment of DEGs

**Figure 3:**
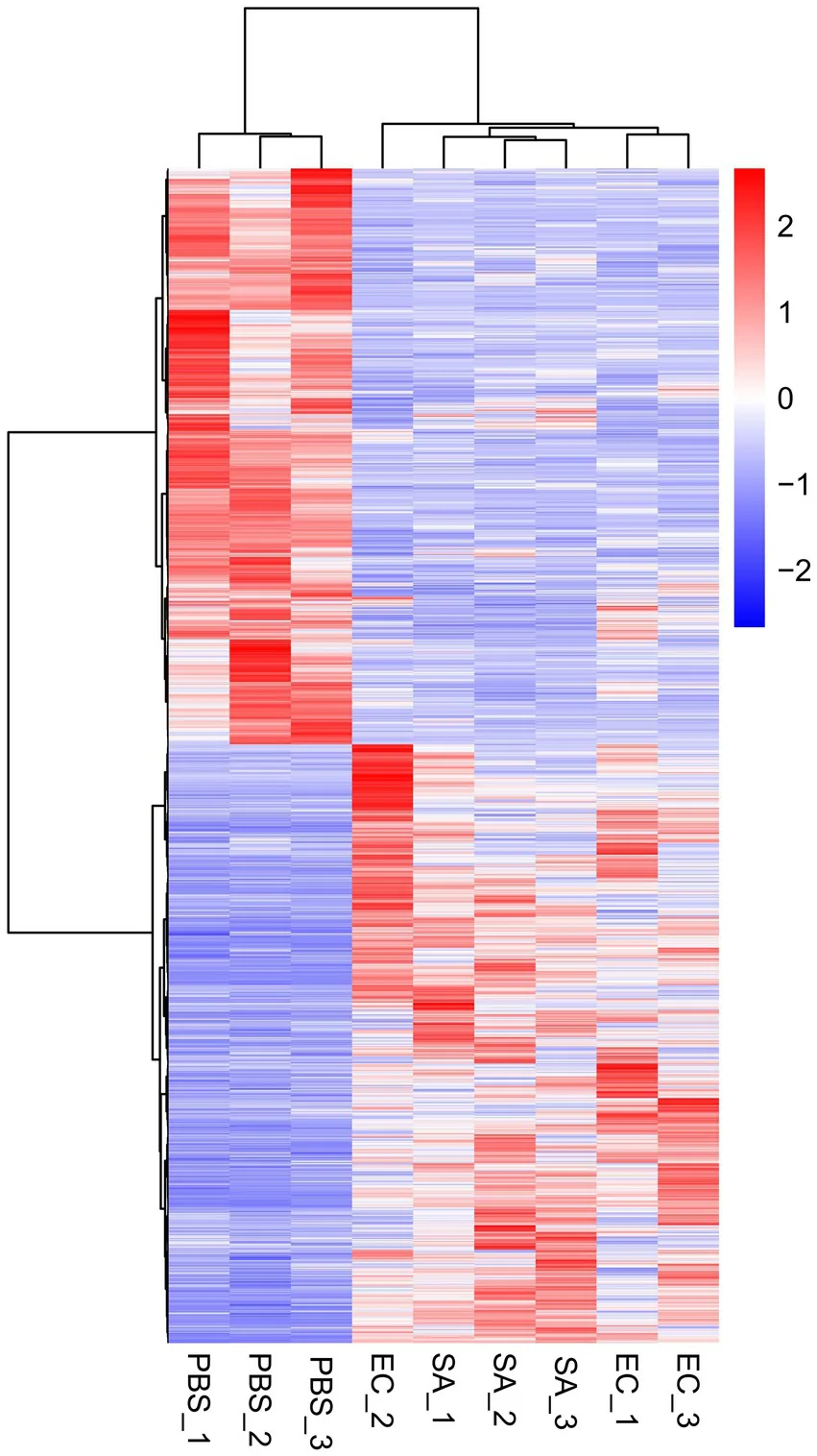
Clustering analysis of differentially expressed genes. K-means clustering of all DEGs across the three experimental groups (PBS, EC, SA). Each row represents a gene, and each column a sample. Expression values are normalized and Z-scored (red: high expression; blue: low expression).

**Figure 4:**
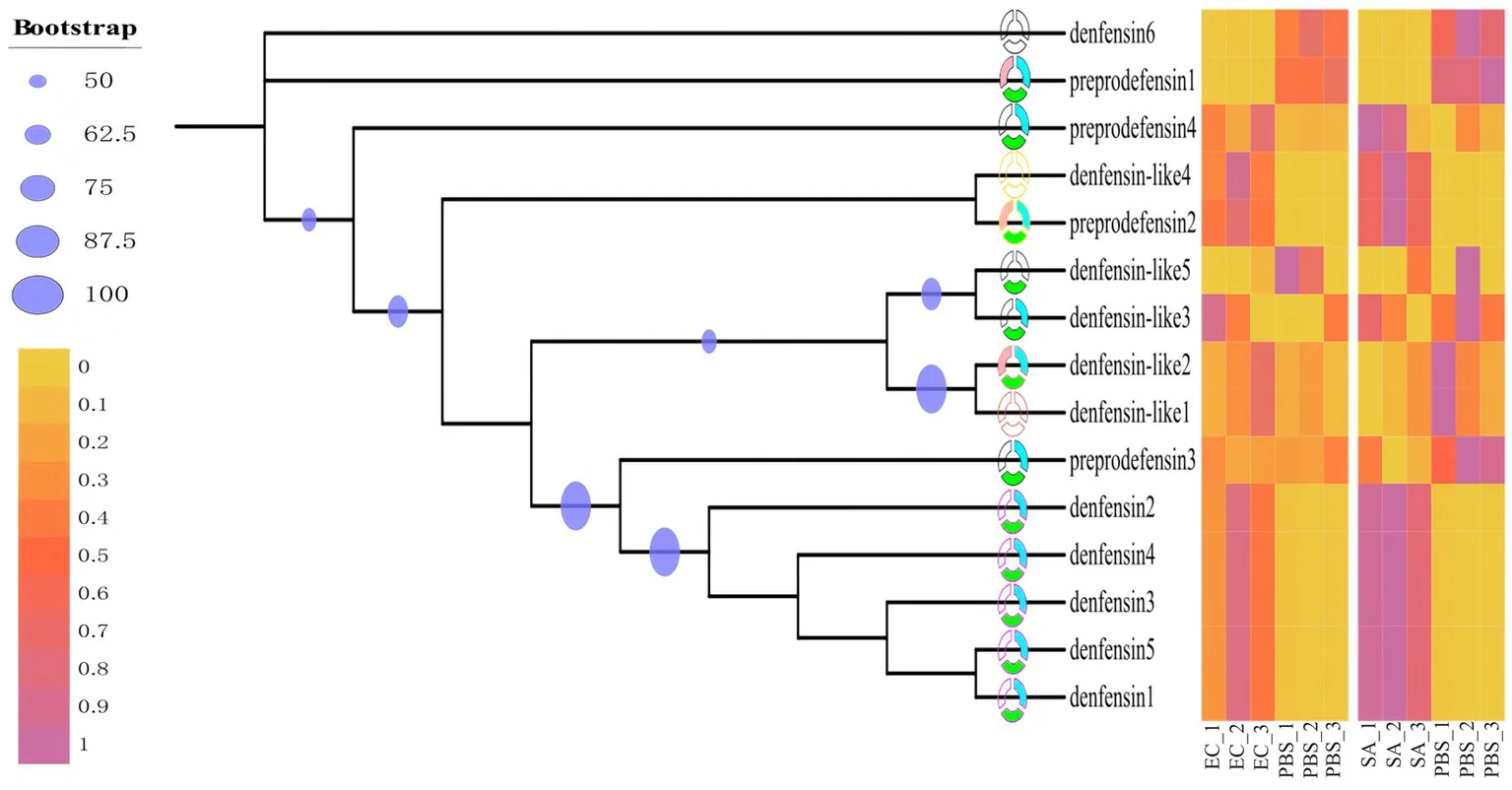
Expression heatmap analysis of defensin genes. Hierarchical clustering of the 15 identified defensin unigenes across PBS, EC, and SA groups. Colour scale represents relative expression levels. Defensin IDs are listed on the right. Several defensin genes were significantly up-regulated in both EC and SA groups compared to PBS controls, while others showed constitutive or down-regulated expression.

K-means clustering of all DEGs delineated distinct expression patterns in response to bacterial challenge (Figure 3). A heatmap analysis demonstrated that a subset of defensin isoforms was significantly up-regulated 24 h post-infection. Notably, defensin isoforms from different phylogenetic clusters exhibited similar expression patterns in both the *E. coli* and *S. aureus* treatment groups, with both up and down-regulated members, indicating a broad and conserved transcriptional response to bacterial challenge rather than a pathogen-specific induction pattern (Figure 4).

**Figure 5:**
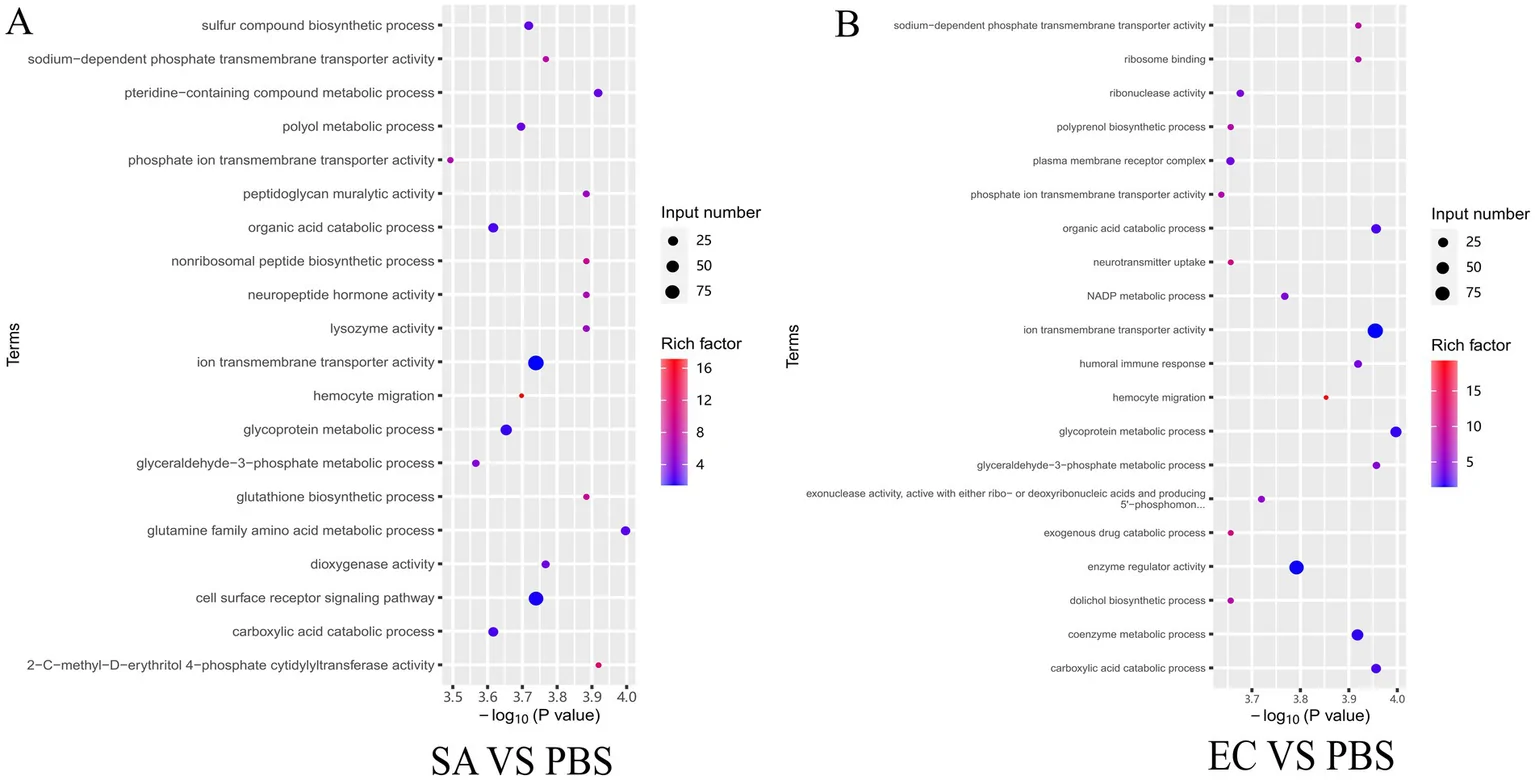
GO enrichment analysis of differentially expressed genes. Top 20 significantly enriched GO terms (biological process, cellular component, and molecular function) for **(A)** SA vs. PBS and **(B)** EC vs. PBS comparisons. Only terms with *p* < 0.05 are shown. The *x*-axis indicates the −log_10_ (*p*-value), and the bar colour represents the enrichment ratio.

Gene Ontology (GO) enrichment analysis of all differentially expressed genes (DEGs) revealed that terms were enriched across all three categories. For the SA/PBS comparison, the five most significantly enriched GO terms were glutamine family amino acid metabolic process, pteridine-containing compound metabolic process, 2-C-methyl-D-erythritol 4-phosphate cytidylyltransferase activity, glutathione biosynthetic process, and nonribosomal peptide biosynthetic process. For the EC/PBS comparison, the five most significantly enriched terms were glycoprotein metabolic process, ion transmembrane transporter activity, organic acid catabolic process, carboxylic acid catabolic process, and glyceraldehyde-3-phosphate metabolic process. Notably, compared with the SA/PBS group, the EC/PBS group exhibited specific enrichment in humoral immune response, suggesting potential differences in the immune pathways activated by the two types of bacterial infection (Figure 5). For visualization, Figure 5 presents the top 20 enriched GO terms according to statistical significance. However, additional immune-associated GO terms identified in the complete enrichment dataset, including immune response, positive regulation of immune system process, positive regulation of immune response, humoral immune response, Toll-like receptor signaling pathway, and JAK–STAT cascade, were also significantly enriched (*p* < 0.05) and are provided in Supplementary Table S5.

**Figure 6:**
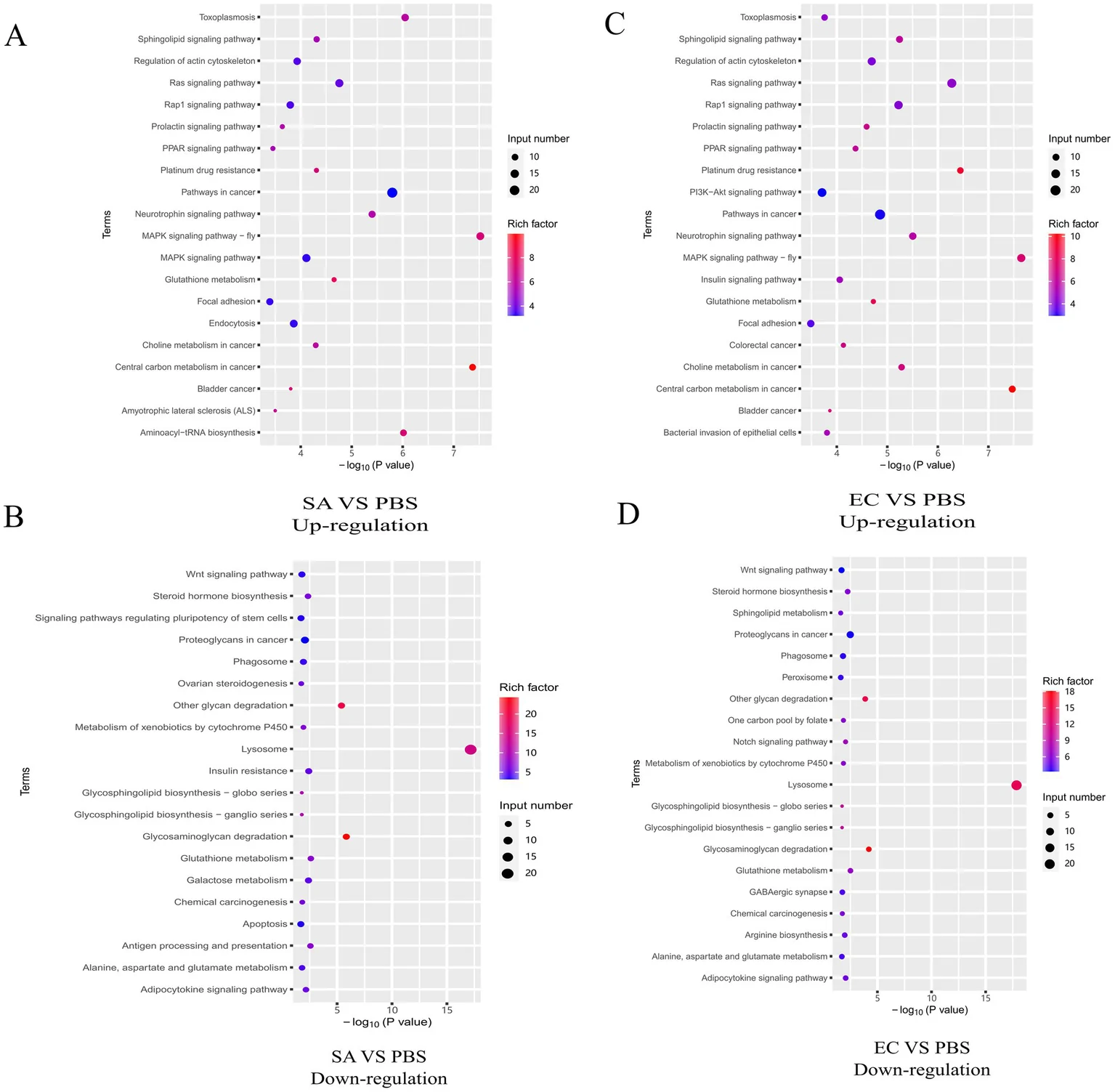
KEGG pathway enrichment analysis of differentially expressed genes. Bubble plots showing significantly enriched KEGG pathways (*p* < 0.05) in **(A)** SA vs. PBS up-regulated DEGs, **(B)** SA vs. PBS down-regulated DEGs, **(C)** EC vs. PBS up-regulated DEGs, and **(D)** EC vs. PBS down-regulated DEGs. The size of each bubble reflects the number of DEGs mapped to the pathway. The colour scale (from blue to red) indicates the magnitude of the rich factor, with red representing higher enrichment.

To gain insight into the potential regulatory networks governing the observed transcriptional changes, KEGG pathway enrichment analysis was performed separately on the up-regulated and down-regulated DEGs in each comparison. In the EC/PBS group, the up-regulated DEGs were significantly enriched in the MAPK signaling pathway, Ras signaling pathway, as well as Toll-like receptor signaling pathway, whereas the down-regulated DEGs were predominantly enriched in metabolic pathways such as Lysosome and Glycosaminoglycan degradation. In the SA/PBS group, the up-regulated DEGs were prominently enriched in the MAPK signaling pathway, Aminoacyl-tRNA biosynthesis, Toll-like receptor signaling pathway and Ras signaling pathway, with the down-regulated DEGs mainly associated with lysosome, Glycosaminoglycan degradation and other glycan degradation (Figure 6 and Supplementary Table S6). Notably, the Lysosome and MAPK signaling pathway was significantly enriched in both treatment groups, suggesting a shared signaling response to Gram-negative and Gram-positive bacterial challenge. These commonly enriched pathways suggest that the two types of bacterial infection may activate the ticks immune response through partially overlapping molecular mechanisms. In particular, the lysosome pathway exhibited high significance among the down-regulated genes in both groups, indicating that this pathway was dramatically regulated upon infection.

A targeted examination of immune-related KEGG pathways further revealed significant enrichment of the Toll-like receptor signaling pathway, the MAPK signaling pathway and the Lysosome pathway (Supplementary Table S6). Within the Toll-like receptor signaling pathway, 8 up-regulated genes were identified as being involved in defense response and Protein kinase domain. The MAPK signaling pathway was affected by 13 up-regulated DEGs, including Putative pleckstrin similarity domain protein and C2H2-type zinc finger. Additionally, 27 DEGs (6 up-regulated and 21 down-regulated) were detected in the Lysosome pathway (Supplementary Table S7). These findings collectively suggest that the Toll-like receptor signaling pathway and the MAPK signaling pathway were predominantly represented by up-regulated genes, suggesting their involvement in immune recognition and signal transduction upon bacterial challenge. In contrast, the lysosome pathway was significantly enriched among down-regulated genes in both treatment groups, pointing to a marked transcriptional suppression of this process following infection. The contrasting expression patterns of these pathways highlight a coordinated shift in transcriptional programs during the antibacterial response of *H. asiaticum*, although the precise functional significance of this regulation awaits further investigation.

### 3.3 Identification and phylogenetic analysis of defensin genes

**Figure 7:**
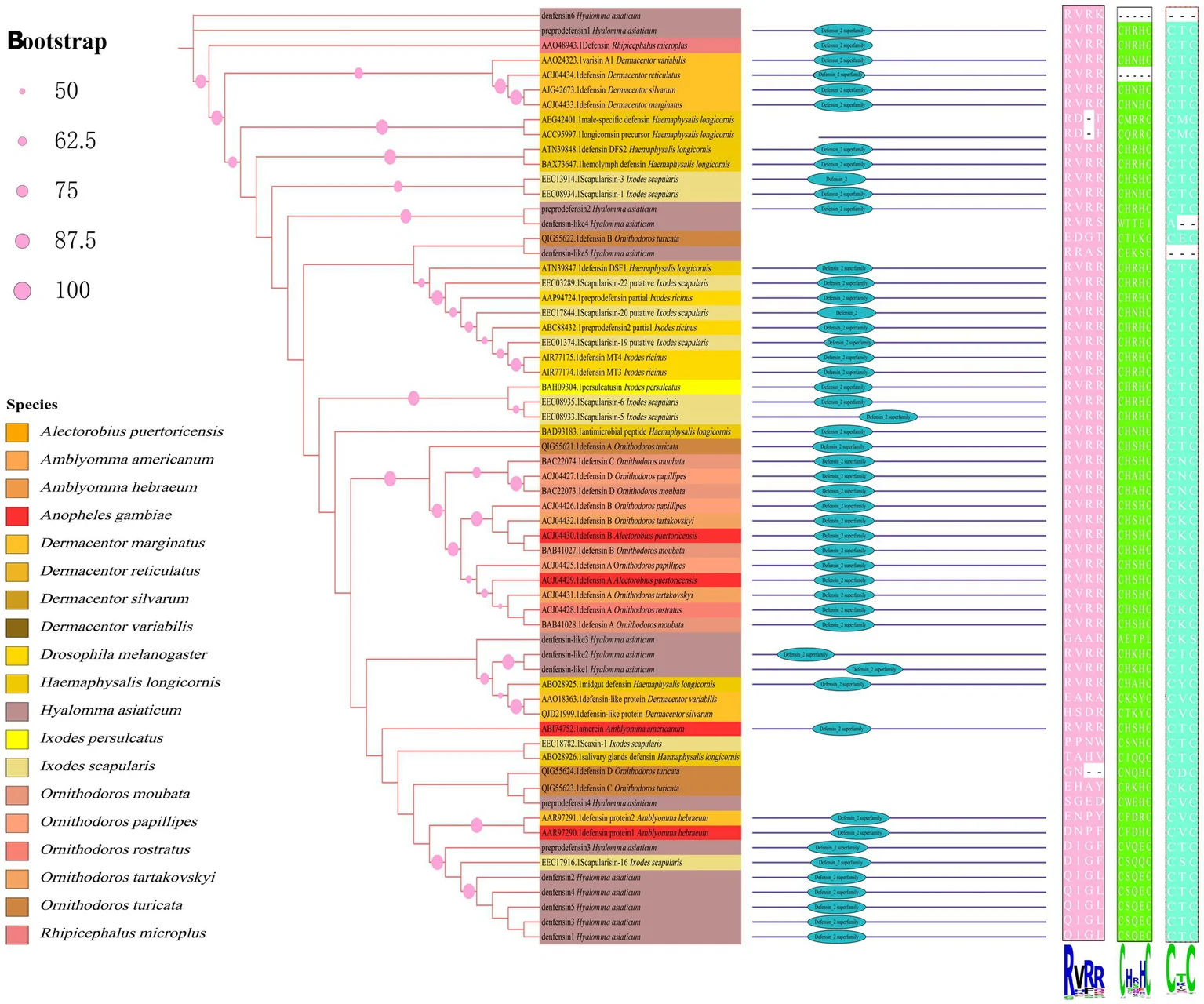
Phylogenetic analysis of defensin genes. Maximum-likelihood phylogenetic tree of 64 defensin sequences from other tick species, including *H. asiaticum* (red branches), *I. scapularis*, *O. turicata*, and *D. marginatus*. The tree was constructed using IQ-TREE2 with the JTT + G4 model and 1,000 ultrafast bootstrap replicates. Multiple sequence alignment of representative defensin precursor proteins showing the conserved RVRR propeptide cleavage site (red box), the CXXXC motif (green box) and the CXC motif (blue box). Alignments were generated with MAFFT and visualized with Jalview.

As antimicrobial peptides are key downstream effectors of innate immune pathways, we next characterized the defensin repertoire of *H. asiaticum*. Through homology-based mining of the *H. asiaticum* transcriptome, we identified 15 putative defensin genes. A maximum-likelihood phylogenetic tree was constructed using these sequences along with reference defensins from 19 other tick species, including *Ixodes scapularis* and *Ornithodoros turicata*. The analysis showed that *H. asiaticum* defensins clustered closely with those from *O. turicata* and *I. scapularis*, demonstrating evolutionary conservation across tick genera (Figure 7). Domain architecture analysis revealed that 49 out of 64 sequences contained the defensin-2 superfamily domain (Pfam: PF01097). Multiple sequence alignment highlighted the high conservation of key motifs, including 37 sequences with the RVRR propeptide cleavage site, and 60 sequences presented disulfide bond formation traits with the CXXXC and CXC motifs (Figure 7).

### 3.4 RT-qPCR validation

**Figure 8:**
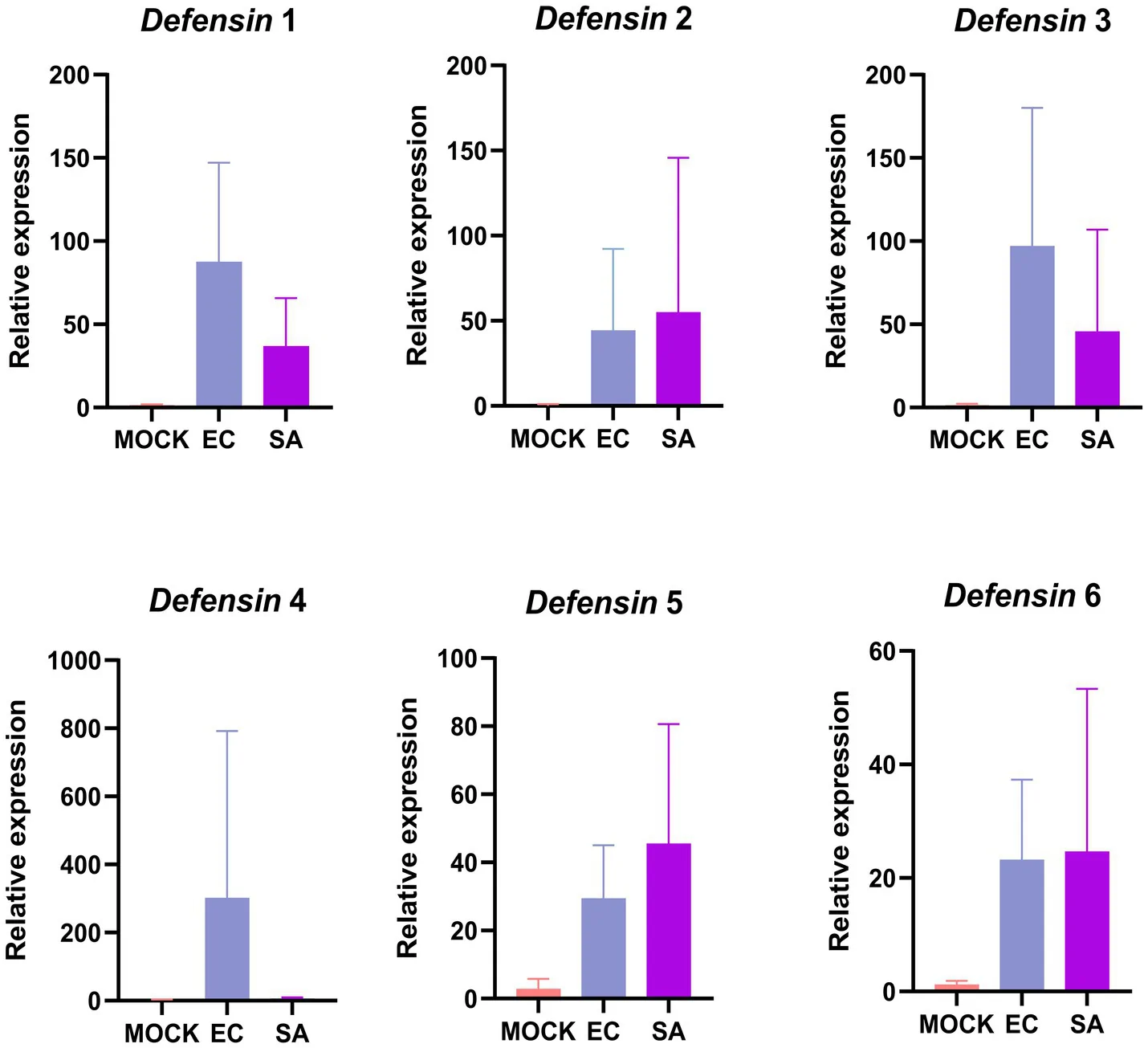
Immunity-related gene expression in infected *H. asiaticum* ticks. *H. asiaticum* translation elongation factor EF-1 alpha (ELF1α) was used as an internal control. Data are presented as mean ± *SEM*. The experiments were repeated three times. Data were normalized to the expression level of ticks from mock-infected group. Mock, PBS-injected females after 24 h; EC, *E. coli*-injected females after 24 h; SA, *S. aureus*-injected females after 24 h.

Selected significantly differentially expressed defensin genes were subjected to RT-qPCR quantification to further validate the transcriptome data. The RT-qPCR results showed expression trends generally consistent with the RNA-seq data. However, due to variability among biological replicates, these differences did not reach statistical significance (*p* > 0.05). These results provide support for the transcriptomic findings but also highlight biological variability in gene expression responses (Figure 8 and Supplementary Table S8).

## 4 Discussion

In this study, a comparative transcriptomic analysis was performed on *H. asiaticum* females challenged with the Gram-negative bacterium *E. coli* and the Gram-positive bacterium *S. aureus*. High-quality RNA-seq data (approximately 386 million clean reads, Q30 > 97%) were generated, and a *de novo* transcriptome assembly yielded 195,132 non-redundant unigenes with an N50 of 843 bp. Functional annotation against six public databases assigned putative functions to 77.8% of unigenes. Principal component analysis (PCA) clearly separated the PBS control, EC and SA groups, indicating distinct transcriptomic reprogramming in response to different bacterial stimuli. Comparative analysis identified 4,294 differentially expressed genes (DEGs) in the EC group and 4,582 DEGs in the SA group compared to the PBS control. Among these, approximately half were up-regulated and half down-regulated in each infection group, suggesting a bidirectional transcriptional reconfiguration rather than a unidirectional activation.

To understand the upstream signaling pathways that govern these effector responses, KEGG pathway enrichment analysis was performed on up-regulated and down-regulated DEGs separately. In the EC/PBS comparison, up-regulated DEGs were significantly enriched in the MAPK signaling pathway, Ras signaling pathway, and Toll-like receptor signaling pathway. In the SA/PBS comparison, up-regulated DEGs were also significantly enriched in the MAPK signaling pathway, Toll-like receptor signaling pathway, Ras signaling pathway and Aminoacyl-tRNA biosynthesis. The MAPK signaling pathway was the most significantly enriched cascade in both infection groups, indicating that it represents a core, shared antibacterial defense mechanism in *H. asiaticum.* The genes involved in this pathway are primarily associated with signal transduction, cell proliferation, differentiation, apoptosis, and immune responses, suggesting that the MAPK cascade plays a central regulatory role in the antibacterial immunity of ticks. This finding is consistent with previous reports in other tick species, suggesting that MAPK activation may be involved in regulating antimicrobial peptide production and phagocytosis, in addition to its well-recognized functions in cell proliferation, differentiation, and apoptosis (8, 9). The Toll-like receptor signaling pathway was significantly enriched in both the EC and SA groups, indicating that it participates in the recognition of both Gram-negative and Gram-positive bacteria in *H. asiaticum*. This differs from the classical view that Toll-like receptors primarily recognize lipopolysaccharide (LPS) from Gram-negative bacteria; ticks may employ a broader recognition spectrum or utilize additional co-receptors for Gram-positive sensing. Such pathway activation patterns suggest that *H. asiaticum* can mount a coordinated immune response against different bacterial classes, while the downstream effector output (defensin up-regulation) is similarly robust (29–31).

Unexpectedly, the lysosome pathway was significantly enriched among down-regulated DEGs in both infection groups. This pathway includes genes involved in phagosomal maturation, acidification, and hydrolytic degradation. Its transcriptional suppression after bacterial challenge may represent a bacterial evasion strategy: both *E. coli* and *S. aureus* have been reported to interfere with host lysosomal function to avoid intracellular killing (32, 33). Alternatively, down-regulation of lysosomal genes could be part of a tick-mediated trade-off, redirecting cellular resources toward rapid production of antimicrobial peptides rather than investing in energetically costly lysosomal turnover. Intriguingly, a similar down-regulation of lysosome-related genes has been reported in *Ixodes scapularis* cells infected with *Anaplasma phagocytophilum*, suggesting that suppression of lysosomal activity might be a conserved vulnerability exploited by multiple tick-borne pathogens (34). However, it is important to acknowledge that these transcriptional changes represent mRNA-level modulation and do not necessarily equate to functional impairment of lysosomal activity. Future studies employing western blotting to quantify lysosomal enzyme protein levels, or enzymatic activity assays to directly measure lysosomal function, are required to determine whether this transcriptional suppression leads to actual functional consequences in *H. asiaticum* immunity. Nevertheless, our transcriptomic data provide a valuable starting point for such mechanistic investigations. It is worth noting that while our PBS control effectively isolates the immune component elicited by bacterial PAMPs from the solvent and general injection stress, the contribution of pure physical injury (e.g., from needle puncture itself) cannot be entirely dissected without a sham-injection control. Nevertheless, the pronounced and pathway-specific enrichment, coupled with the massive up-regulation of defensins far exceeding typical wound-response levels, strongly suggests that the differential pathway engagement is driven predominantly by bacterial challenge. Future work incorporating mock-injury group controls will allow for more information on the injury-versus-infection signals in tick immunity.

A key goal of this study was to identify and characterize defensin genes, which are major effector molecules of tick innate immunity. Through homology-based mining, 15 putative defensin sequences were identified in the *H. asiaticum* transcriptome. To comprehensively analyze sequence characteristics, the 15 *H. asiaticum* defensin sequences were combined with defensin homologs retrieved from 19 other tick species (including *Ixodes scapularis*, *Ornithodoros turicata*, and *Dermacentor marginatus*), resulting in a total of 64 defensin sequences for structural and phylogenetic analysis. Phylogenetic analysis revealed that *H. asiaticum* defensins cluster closely with homologs from *O. turicata* and *I. scapularis*, suggesting strong evolutionary conservation across tick genera (Figure 7). This conserved phylogeny aligns with the hypothesis of convergent evolution in invertebrate defensin superfamilies (35). Notably, 49/64 defensin sequences contained the defensin-2 superfamily domain (Pfam: PF01097), while 15 lacked this domain, indicating potential functional diversification (Figure 7). Domain truncation or loss may reflect adaptive specialization, such as tissue-specific activity or novel antimicrobial targets (36). For instance, defensins lacking canonical domains may mediate non-classical roles in immune modulation or host-pathogen coadaptation (23, 37). Previous studies have revealed three critical features of conserved defensin motif: the RVRR propeptide cleavage site (37/64 sequences), essential for releasing mature peptides from precursor proteins (38); the CXXXC motif (62/64) stabilizing the *α*-helix; and the CXC motif (61/64) anchoring *β*-strands via disulfide bonds (Figure 7). These motifs collectively maintain the CSαβ scaffold, a hallmark of invertebrate defensins (39, 40). The expression of tick defensins is known to exhibit tissue-specific patterns, with transcripts detected in the midgut, salivary glands, hemolymph and ovary depending on the species and physiological state (18, 20, 41). In *Ixodes pacificus*, for example, certain defensin isoforms are abundantly expressed in the midgut of unfed ticks. However, the use of whole-tick homogenates, rather than dissected tissues, precluded the resolution of the spatial distribution of the 15 defensin isoforms identified here. Consequently, it remains unclear whether the observed transcriptional up-regulation following bacterial challenge occurs ubiquitously across all tick tissues or is restricted to specific organs such as the midgut or salivary glands. Future studies employing tissue-specific expression profiling would be valuable to address this question.

Although a previous transcriptomic study on *H. asiaticum* infected with the fungus Beauveria bassiana and the Gram-negative bacterium *Enterobacter cloacae* identified 68 potential immunity-related genes and described the presence of Toll and JAK/STAT pathway components (31), the present work extends this foundation in several important directions. Instead of relying solely on homology-based annotation, this study systematically characterized the defensin repertoire of *H. asiaticum*, identifying 15 putative defensin genes and analyzing their phylogenetic relationships and conserved motif structures (RVRR, CXXXC, CXC), which represents the first comprehensive defensin family analysis in this species. Our comparative challenge design using both Gram-negative (*E. coli*) and Gram-positive (*S. aureus*) bacteria further revealed that *H. asiaticum* mounts a coordinated transcriptional response to both types of bacteria, with defensin up-regulation as a common and robust effector output. More notably, the lysosome pathway was significantly down-regulated following bacterial challenge in both infection groups, a finding previously unreported in *H. asiaticum* that potentially reflects a metabolic trade-off redirecting cellular resources toward antimicrobial peptide production. Collectively, these novel aspects provide a more detailed view of the antibacterial immune response in *H. asiaticum*, with particular emphasis on effector molecules (defensins) and previously unrecognized transcriptional modulation (lysosome suppression).

Based on our transcriptomic analysis, defensin genes were significantly up-regulated in both EC and SA treatment groups, suggesting that *H. asiaticum* defensins act as broad-spectrum antimicrobial effectors rather than being strictly pathogen-specific. This consistent and marked induction further suggests that defensins may contribute to the tick’s immune response beyond their direct antimicrobial activity. In addition, the global set of DEGs was significantly enriched in pathways governing apoptosis and oxidative stress responses. Although these pathway enrichments do not prove a direct causal relationship, they raise the possibility that defensins might participate in the regulation of programmed cell death-potentially to eliminate infected or damaged cells and may also aid in counteracting oxidative stress triggered by bacterial invasion (42). If confirmed, such multifunctional roles would position defensins as key integrators of antimicrobial defense, tissue homeostasis, and stress adaptation in *H. asiaticum*. While this potential strategy remains to be fully characterized in ticks, similar phenomena have been observed in other arthropods, such as in fruit flies and mosquitoes, where defensin expression has been associated with oxidative stress responses and immune modulation (42, 43). For instance, certain defensin isoforms might contribute to balancing pathogen defense with metabolic homeostasis by binding reactive oxygen species (ROS) during prolonged blood-feeding (14). Therefore, these transcriptomic findings offer new clues regarding the diverse potential functions of defensins in tick immunity beyond their traditional antimicrobial roles, necessitating further functional biological experiments to validate their specific roles and regulatory mechanisms in programmed cell death and oxidative stress (44).

Defensins may contribute to host-microbe coexistence through their molecular specificity. Evolutionary pressure likely favors defensin isoforms that potently neutralize common environmental opportunistic pathogens while preserving the persistence and transmission of obligate intracellular bacteria (6, 45). Such functional specialization is critical for maintaining the tick’s vector competence (19).

The study provides a foundational transcriptomic resource and reveals key immune features of *H. asiaticum* using a laboratory population, which minimizes genetic variability and environmental noise. The conserved defensin motifs and core immune pathways identified here are likely fundamental across populations. However, the generalizability of the quantitative expression dynamics and potential existence of population-specific defensin isoforms warrant further investigation in ticks from diverse geographical origins, which may have experienced distinct pathogen-driven evolutionary pressures.

## 5 Conclusion

This study characterizes the defensin repertoire and immune transcriptomic response of *H. asiaticum* to Gram-negative *E. coli* and Gram-positive *S. aureus* bacteria. We identified 15 putative defensin genes in the *H. asiaticum* transcriptome, several of which were significantly up-regulated following bacterial challenge, indicating their involvement in the antibacterial response. KEGG pathway analysis revealed that the MAPK and Toll-like receptor signaling pathways were commonly activated in response to both types of bacteria. In contrast, the lysosome pathway was significantly down-regulated in both infection groups. These findings provide a transcriptomic resource for understanding tick innate immunity and highlight candidate defensins and signaling pathways that may serve as targets for interrupting tick-borne pathogen transmission.

## Data Availability

The datasets presented in this study can be found in online repositories. The names of the repository/repositories and accession number(s) can be found at: NCI, accession number GSE319477; https://www.ncbi.nlm.nih.gov/nuccore/?term=GSE319477.

## Footnotes

1
http://www.genome.jp/tools-bin/clustalw

2
http://itol.embl.de

3
https://www.ncbi.nlm.nih.gov/geo/query/acc.cgi?acc=GSE319477
