# Supplementary material for: Comparative analysis of defensin expression in Hyalomma asiaticum ticks exposed to Staphylococcus aureus and Escherichia coli
Source: Front Vet Sci. 2026 Aug 24;13:1902523. doi: 10.3389/fvets.2026.1902523 (PMC13548987; doi:10.3389/fvets.2026.1902523)
Supplement: Supplementary file 1 [file Supplementary_file_1.zip › Supplementary Material 1/Supplementary Images 1 and 2, Supplementary Tables 1 and 2.DOCX]

# *Supplementary Material*

# 2 Supplementary Figures and Tables

**2.1** Supplementary Figures

## Supplementary Figure 1. Gene Ontology (GO) functional classification statistics of the *H. asiaticum* transcriptome. The bar chart shows the number of unigenes assigned to biological process (BP), cellular component (CC), and molecular function (MF) categories.

**Supplementary Figure 2. KEGG pathway category distribution of annotated unigenes from the *H. asiaticum* transcriptome.** The bar chart shows gene counts across six main categories: Metabolism, Genetic Information Processing, Environmental Information Processing, Cellular Processes, Organismal Systems, and Human Diseases.

## 2.2 Supplementary Tables

**Supplementary Table 1.** The database of known defensins of ticks.

| **NCBI accession number** | **Tick species** | **Sequence information** |
| --- | --- | --- |
| BAB41027.1 | *Ornithodoros moubata* | MNKLFIVALVVALAVATMAQEVHDDVEEQSVPRVRRGYGCPFNQYQCHSHCRGIRGYKGGYCTGRFKQTCKCY |
| BAB41028.1 | *Ornithodoros moubata* | MNKLFIVALVVALAVATMAQEVHNDVEEQSVPRVRRGYGCPFNQYQCHSHCSGIRGYKGGYCKGTFKQTCKCY |
| BAC22074.1 | *Ornithodoros moubata* | MNKLFIVALVLALAVATMAHEVYDDVEEPSVPRVRRGYGCPFNQYQCHSHCSGIRGYKGGYCKGLFKQTCNCY |
| BAC22073.1 | *Ornithodoros moubata* | MNKLFIVALVLALAVATMAHEVHDDIEEPSVPRVRRGFGCPFNQYECHAHCSGVPGYKGGYCKGLFKQTCNCY |
| ACJ04425.1 | *Ornithodoros moubata* | MNKLFIVALVVALAVATMAQEVHDDVEEQSVPRVRRGYGCPFNQYQCHSHCSGIRGYKGGYCKGTFKQTCKCY |
| ACJ04426.1 | *Ornithodoros moubata* | MNKLFIVALVVALAVATMAQEVHDDVEEQSVPRVRRGYGCPFNQYQCHSHCRGIRGYKGGYCTGRFKQTCKCY |
| ACJ04427.1 | *Ornithodoros moubata* | MNKLFIVALVLALAVATMAHEVHDDIEEPSVPRVRRGFGCPFNQYECHAHCSGVPGYKGGYCKGLFKQTCNCY |
| ACJ04429.1 | *Alectorobius puertoricensis* | MNKLFIVALVAALAVATMAQEVHNDVEEQSVPRVRRGYGCPFNQYQCHSHCSGIRGYKGGYCKGTFKQTCKCY |
| ACJ04430.1 | *Alectorobius puertoricensis* | MNKLFIVALVVALAVATMAQEVHDDVEEQSVPRVRRGYGCPFNQYQCHSHCRGIRGYKGGYCTGRFKQTCKCY |
| ACJ04428.1 | *Ornithodoros rostratus* | MNKLFIVALVVAPAVATMAQEVHNDVEEQSVPRVRRGYGCPFNQYQCHSHCSGIRGYKGGYCKGTFKQTCKCY |
| ACJ04431.1 | *Ornithodoros tartakovskyi* | MNKLFIVALVVALAVATMAQEVHNDVEEQSVPRVRRGYGCPFNQYQCHSHCSGIRGYKGGYCKGTFKQTCKCY |
| ACJ04432.1 | *Ornithodoros tartakovskyi* | MNKLFIVALVVALAVATMAQEVHDDVEEQSVPRVRRGYGCPFNQYQCHSHCRGIRGYKGGYCTGRFKQTCKCY |
| QIG55621.1 | *Ornithodoros turicata* | MKTVFVIALVFALAVASMAQDVDDVEESSAVRVRRGYGCPFNQYQCHSHCSGIRGYKGGYCKGLFKQTCTCY |
| QIG55622.1 | *Ornithodoros turicata* | MKVLCFLLLLLLTGLLTSRAAVLDTRRDPEDGTGNDCPHNEIACTLKCERDGFAYGRCTGLVLDQKCECIA |
| QIG55623.1 | *Ornithodoros turicata* | MTPLRFSLVCFLVLSAVVTATAFQLRSVHNTEHAYGCPGYRAMCRKHCVETFGFEGYCGGAHRNECKCRG |
| QIG55624.1 | *Ornithodoros turicata* | MKIVLVLLVCVMAFGVHSSPPAADGGSGYGNGCPSNPAQCNQHCLDTRDLTGHCKGYQMTFCDCGW |
| ABI74752.1 | *Amblyomma americanum* | MKVLAVAFIFVLVAGLVSTADEEDKSQVPLVRVRRGFGCPFNQYQCHSHCLSIGRRGGYCGGSFKTTCTCYN |
| AAR97290.1 | *Amblyomma hebraeum* | MATVRNSRPEAAGEPSGVSSTEGDWRHIEKRDVSYQGEGNTRRFDNPFGCPADEGKCFDHCNNKAYDIGYCGGSYRATCVCYRK |
| AAR97291.1 | *Amblyomma hebraeum* | MATQRREISWTFGPLYTWRTTKGYGTTLETTNATSTSSKPSRRYENPYGCPTDEGKCFDRCNDSEFEGGYCGGSYRATCVCYRT |
| ACJ04433.1 | *Dermacentor marginatus* | MRGLCICLVFILVCGLLTATAAVPAESEAAHLRVRRGFGCPLNQGACHNHCRSIRRRGGYCSGIIKQTCTCYRN |
| ACJ04434.1 | *Dermacentor reticulatus* | MRGLCICLVFILVCGLLTATAAVPAESEAAHLRVRRGGYCSGIIKQTCTCYRN |
| AJG42673.1 | *Dermacentor silvarum* | MRGLCICLVFILVCGLLTATAAVPAESEAAHLRVRRGFGCPLNQGACHNHCRSIRRRGGYCSGIIKQTCTCYRN |
| QJD21999.1 | *Dermacentor silvarum* | MKFTSAIFLIAVLAAFLAMMTAAEESSENMGGAHSDRGGCPDADKCTKYCQKQGISVGKCAKPVNACVCIL |
| AAO24323.1 | *Dermacentor variabilis* | MRGLCICLVFLLVCGLVSATAAAPAESEVAHLRVRRGFGCPLNQGACHNHCRSIRRRGGYCSGIIKQTCTCYRN |
| AAO18363.1 | *Dermacentor variabilis* | MRFTSAIFLVAVIAAFVVMITATGERSEERSEEARASGCKADACKSYCKSLGSGGGYCDQGTWCVCN |
| BAD93183.1 | *Haemaphysalis longicornis* | MKVLAVALIFVLVAGLFCTAAAQDDESDVPHVRVRRGFGCPLNQGACHNHCRSIGRRGGYCAGIIKQTCTCYRK |
| ABO28925.1 | *Haemaphysalis longicornis* | MKVFSALFLVGLLLAFLAFAAGDEEDSSKPLVRVRRGFGCPFDERACHAHCQSVGRRGGYCGNFRMTCYCYKN |
| ABO28926.1 | *Haemaphysalis longicornis* | MKLLVVCAALTVLGGLLQGATCDDSDHGFRTAHVDLVCPDNPDNCIQQCVSKGAQGGYCTNEKCTCYEKIPSATKRVRIVA |
| ACC95997.1 | *Haemaphysalis longicornis* | MAESTTTCFLLLVTGYVTAVMSEEAHLRSRRDFGCGQGMIFMCQRRCMRLYPGSTGFCRGFRCMCDTHIPLRPPFMVG |
| AEG42401.1 | *Haemaphysalis longicornis* | MARSNLLLLLLLAAVTIVAVAEEAAHHRARRDFGCARGMIFVCMRRCARMYPGSTGYCQGFRCMCDTHIPIRRPPFIMG |
| BAX73647.1 | *Haemaphysalis longicornis* | MRVLVLSLAVILLCGLMAGSATAEEESEVAHLRVRRGFGCPLNQGACHRHCRSIRRRGGYCSGIIKQTCTCYRN |
| ATN39847.1 | *Haemaphysalis longicornis* | MKLLAVVLALVLVIGAVAKGAVTEEKGEVAHLRVRRWGFGCPFNARRCHRHCRSIRRRAGYCAGRLRLTCTCVR |
| ATN39848.1 | *Haemaphysalis longicornis* | MRVLVLSLAVILLCGLMAGSATAEEESEVAHLRVRRGFGCPLNQGACHRHCRSIRRRGGYCSGIIKQTCTCYRN |
| BAH09304.1 | *Ixodes persulcatus* | MRVVAVALIALLVAGAFMTSSAQEEENQVAHVRVRRGFGCPFNQGACHRHCRSIGRRGGYCAGLFKQTCTCYSR |
| AAP94724.1 | *Ixodes ricinus* | MKVLAVSLAFLLIAGLISTSLAQNEEGGEKELVRVRRGGYYCPFFQDKCHRHCRSFGRKAGYCGGFLKKTCICVMK |
| ABC88432.1 | *Ixodes ricinus* | MKVLAVSLAFLLIAGLISTSLAENDEGGEKELVRVRRGGYYCPFRQDKCHRHCRSFGRKAGYCGGFLKKTCICV |
| AIR77174.1 | *Ixodes ricinus* | MKVLAVSLAFLLIAGLITSSLAENDEGGEKELVRVRRGGYYCPFRQDKCHRHCRSFGRKAGYCGNFLKRTCICVKK |
| AIR77175.1 | *Ixodes ricinus* | MKVLAVSLAFLLIAGLITTSLAENDEGGEKELVRVRRGGYYCPFRQDKCHRHCRSFGRKAGYCGNFLKRTCICVKK |
| EEC08934.1 | *Ixodes scapularis* | MKAVAIALVVMMIAGLISTSCSQEDDSQVAHVRVRRGFGCPLNQGACHNHCRSIKRRGGYCSGIIKQTCTCYRK |
| EEC13914.1 | *Ixodes scapularis* | MKVVGIALVVRLFSFSCSQGVHSQVPHVRVRRAFGCPFDQGTCHSHCRSIRRRGERCSGFAKRTCTCYQK |
| EEC08933.1 | *Ixodes scapularis* | MDTTATFKLSSTKNTVQVEHRIINLEIMKVIAVALIALLVAGAFMTSSAQEEEDQVAHVRVRRGFGCPFDQGACHRHCQSIGRRGGYCAGIIKQTCTCYHN |
| EEC08935.1 | *Ixodes scapularis* | MRVIAVTLIALLVAGAFMTSSAQEEENQVAHVRVRRGFGCPFDQGACHRHCQSIGRRGGYCAGFIKQTCTCYHN |
| EEC17916.1 | *Ixodes scapularis* | MNTSSLFTVALIAFAGFMTIQLVSPYVIQPFFDIGFGCPKSALSCSQQCRENNTHSGGYCNGPFNIVCSCY |
| EEC01374.1 | *Ixodes scapularis* | MKVLAVSLAFLLITGLISTSLAENDEGGEKELVRVRRTSYNCPFQKHKCHRHCKSIGHIAGYCGGFRNRTCICVKK |
| EEC17844.1 | *Ixodes scapularis* | MKVLAVPLAFLLVVGLISTSLAQNGEGGEKELVRVRRGGYYCPFRQDICHNHCRSFGRKAGYCGGFLKKTCICVMK |
| EEC03289.1 | *Ixodes scapularis* | MKVLAVSLAFLLIAGLISTSFAQNEEGGEKELVRVRRGGYYCPFFQDKCHRHCLSIGRRAGYCGGFLKKTCICVMK |
| EEC18782.1 | *Ixodes scapularis* | MAALRFTLFALVAVSVASAGYFHIEDKPGPPNWGCPELEIACSNHCFSIGCRIGECRNFPFFTDCTCLNPI |
| AAO48943.1 | *Rhipicephalus microplus* | MRGIYICLXFVLXCGLVSGLADVPAESEMAHLRVRRGFGCPFNQGACHRHCRSIRRRGGYCAGLIKQTCTCYRN |

**Supplementary Table 2.** Specific Primers and Amplification System for RT-qPCR Validation of RNA-seq Results.

| **Primer pairs** | **(5’ to 3’ end)** | **Anotation** | **PCR Product** | **Amplification system** |
| --- | --- | --- | --- | --- |
| Defensin1-RT-F  Defensin1-RT-R | TAGCCGTTCATGTGCCTGTA  CGAGCTTCATCCACACACAG | Defensin | 110 | 20 μl reaction, 2 × qPCR Mix 10 μl, 1μl each for forward and reverse primers, 1 μl of sample cDNA, 7 μl of ddH_2_O. |
| Defensin2-RT-F  Defensin2-RT-R | TTCTCGAGGTGCTCTTCCTG  GTTCACCCAAGCACGATGTT | Defensin | 128 |  |
| Defensin3-RT-F  Defensin3-RT-R | GACAATCAGTTCCGCATCCC  CACGCGCACAAATGTCTTG | Defensin | 119 |  |
| Defensin4-RT-F  Defensin4-RT-R | GTCGATTTCGCGAGGTACAG  AATCTGAACGGCGAACACAG | Defensin | 124 |  |
| Defensin5-RT-F  Defensin5-RT-R | TTAACTCGCGGCATCAAAGG  ATCGTCGACGTCATCTCCAA | Defensin | 117 |  |
| Defensin6-RT-F  Defensin6-RT-R | CCCGGGTGTAACAATATCGC  GCTGGTGGACGAAACACTTT | Defensin | 117 |  |
| Ef-1α-RT-F  Ef-1α-RT-R | CCTGCCAACCTGACCACTGA  CCACACACATATCCTCGGCG | Elongation factor 1 alpha | 137 |  |

**Supplementary Table 3. Annotation table of *H. asiaticum* transcriptome analysis.** Upload as an EXCEL table separately.

**Supplementary Table 4. KEGG pathway annotation statistics of *H. asiaticum.*** Upload as an EXCEL table separately.

**Supplementary Table 5.** Detailed Statistical Results of GO Enrichment Analysis for Differentially Expressed Genes. Upload as an EXCEL table separately.

**Supplementary Table 6**. Detailed Statistical Results of KEGG Pathway Enrichment Analysis for Differentially Expressed Genes. Upload as an EXCEL table separately.

**Supplementary Table 7.** Tables of Differentially Expressed Genes in the Toll-like receptor signaling pathway, the MAPK signaling pathway and the Lysosome pathway. Upload as an EXCEL table separately.

**Supplementary Table 8. Functional annotation and expression levels of defensin genes with differential expression.** Upload as an EXCEL table separately.
